# Management of a “Can Intubate, Can’t Ventilate” Airway Post-tracheostomy: A Case Report

**DOI:** 10.7759/cureus.115410

**Published:** 2026-08-29

**Authors:** Roy Madrid, Sergio Alvarado Gil, Lorenzo E Juarez, Hemza Assed, Eugene Koshkin

**Affiliations:** 1 Department of Medical Education, University of New Mexico School of Medicine, Albuquerque, USA; 2 Department of Anesthesiology and Critical Care Medicine, University of New Mexico School of Medicine, Albuquerque, USA; 3 Department of Anesthesiology and Critical Care Medicine, University of New Mexico Hospital, Albuquerque, USA

**Keywords:** airway, bronchoscope, case report, intubation, tracheostomy, ventilation

## Abstract

In this case report of a 36-year-old with a remotely healed tracheostomy stoma, urgent intubation led to false passage formation despite the endotracheal tube appearing to pass through the vocal cords. Absent end-tidal CO₂ and breath sounds were initially attributed to severe bronchospasm, but progressively increasing subcutaneous air prompted fiberoptic bronchoscopy, which revealed a paratracheal false passage. The patient developed hypoxic cardiac arrest, required extracorporeal membrane oxygenation, and, ultimately, died. This case underscores the critical need for early bronchoscopy evaluation when standard confirmation methods are inconclusive, as well as the importance of avoiding anchoring bias in airway emergencies.

## Introduction

Tracheostomy is a common surgical procedure in the United States, with an estimated incidence of 34.3 cases per 100,000 adults annually [1]. Anesthesiologists often anticipate tracheal narrowing at the site of a prior tracheostomy; however, a rare but severe complication that can occur is false passage formation during intubation [2]. Fiberoptic bronchoscopy (FOB) is an essential tool for managing difficult airways, offering precise visualization and guidance for endotracheal tube (ETT) placement in complex cases [3]. In patients with a history of tracheostomy, complications such as false-passage airways are underrecognized, particularly when the tracheostomy site appears healed. Early use of FOB may facilitate rapid assessment and correction of airway issues, potentially avoiding adverse outcomes, but their use has not been consistently integrated into airway algorithms [3,4].

When severe complications occur, management may be further complicated by cognitive biases that can influence clinical decision-making [5,6]. Studies have shown that cognitive biases can lead to incorrect or delayed diagnoses, resulting in adverse outcomes [7]. Anchoring bias, i.e., fixating on an initial diagnosis or assumption, can delay consideration of alternative explanations, particularly during time-sensitive situations such as airway emergencies [6]. In anesthesiology, where seconds can make a crucial difference, awareness of such biases is crucial. Although literature regarding cognitive errors in anesthesia is limited, a comparative study found that delayed diagnosis of high neuraxial block (high spinal) was a significant preventable cause of maternal brain damage [8].

Sparse literature exists on ETT placement in patients with a history of tracheostomy, and strategies to prevent false passage formation are not well documented in current difficult airway algorithms [4]. This case presents a false-passage airway in a presumptively healed tracheostomy stoma, emphasizing the importance of prompt access to FOB for airway evaluation and recognizing cognitive biases that may influence clinical decisions. Together, these insights highlight the need for both advanced tools and heightened vigilance to optimize outcomes in challenging airway scenarios.

The case was discussed with the patient’s legally authorized next of kin, who provided verbal agreement for publication of this case report. All data were anonymized, and all potentially identifying information was removed. This article was presented as a meeting abstract at the 2024 Annual Anesthesiology Meeting on October 19, 2024.

## Case presentation

A 36-year-old female was recently discharged from an outside hospital to a long-term acute care (LTAC) hospital following a two-month intensive care unit (ICU) stay after exploratory laparotomy for a gunshot wound with associated hemorrhagic shock, renal artery laceration, and abdominal aortic dissection. She was admitted from the LTAC due to suspected bowel ischemia with CT evidence of pneumatosis, a rising white blood cell count to 18.8 × 10³/µL, and an elevated lactate of 2.2 mmol/L.

Upon admission, she had a non-healing midline abdominal wound managed with a wound vacuum and multiple drains. She measured 140 cm and weighed 73 kg, with a BMI of 37.2 kg/m². Physical examination revealed a loose Telfa dressing on her neck, intended to “protect” a previous tracheostomy scar from the hospitalization two months prior. The site appeared externally healed, and the dressing was replaced. Although no decannulation date was known, record review indicated that at least two weeks had passed since admission to the LTAC. A chest X-ray from two weeks prior (Figure 1) showed a mild right hemithorax without other acute abnormalities, and she was scheduled for urgent surgery.

**Figure 1:**
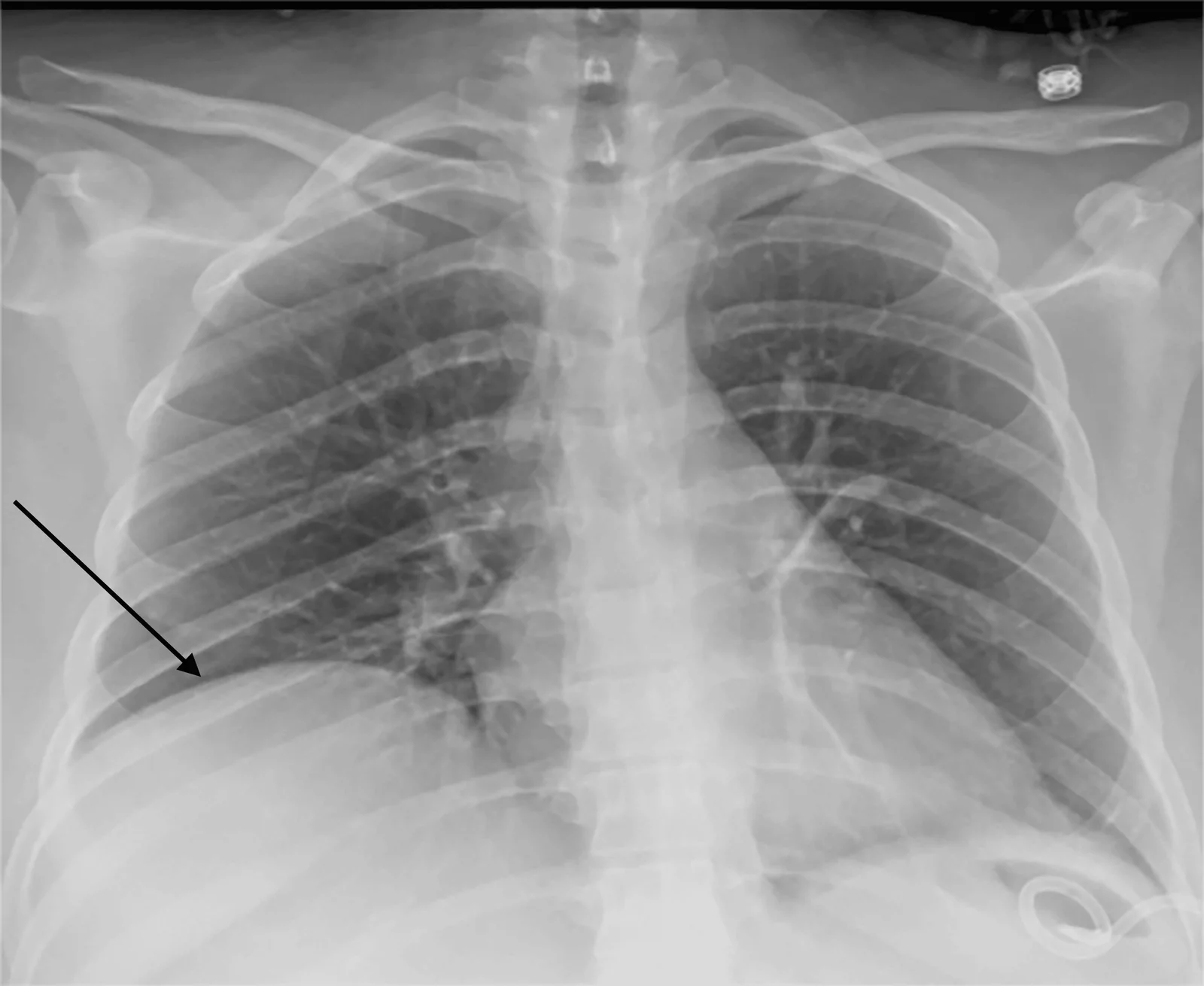
Preoperative anteroposterior chest X-ray. Preoperative chest X-ray showed only mild right hemithorax (arrow) without acute findings.

She was intubated with a direct Macintosh laryngoscope, which provided a full view (Cormack-Lehane grade 1) of the vocal cords. The ETT was visualized past the vocal cords and secured at 21 cm at the teeth. Despite the ETT passing between the vocal cords, there was absent end-tidal CO₂, increased airway resistance, and high inspiratory pressures with absent bilateral breath sounds due to suspected bronchospasm. Within the next five minutes, she had worsening oxygen saturation with inadequate ventilation despite appropriate intubation and positioning; hence, a code was called with immediate initiation of cardiopulmonary resuscitation using advanced cardiovascular life support protocols and treatment for presumed bronchospasm.

Ventilation treatment for presumed bronchospasm remained difficult with no signs of end-tidal CO₂ despite high peak airway pressures. Due to inadequate ventilation over the next few minutes, the extracorporeal membrane oxygenation (ECMO) team was called, who completed cannulation within 15 minutes and achieved effective circulation shortly thereafter.

Ultimately, the team noticed swelling in the lower neck, face, and chest, suggestive of subcutaneous air leakage from the airway. The dressing over the previous tracheostomy site was removed, revealing a sealed stoma. Manual palpation of the neck raised suspicion of subcutaneous placement of the ETT. Visualization with an FOB showed only pink tissue. Suctioning did not improve FOB visualization distal to the ETT tip. An incision was made at the old tracheostomy site, and a Shiley tracheostomy tube was inserted as the ETT was retracted. The anesthesia circuit was then connected to the Shiley tracheostomy tube, resulting in successful ventilation with detection of end-tidal CO₂, confirming appropriate airway placement.

Following surgery, the patient was transferred to the ICU. CT of the chest, abdomen, and pelvis with angiography revealed right middle and lower lobe aspiration pneumonia, pneumomediastinum, moderate left pneumothorax, and significant soft-tissue emphysema in the chest, thoracic inlet, and neck (Figure 2). These findings suggested a false passage resulting in an extremely rare “can intubate, can’t ventilate” scenario [9,10]. The patient was transferred to the ICU, developed diffuse hypoxic-ischemic brain injury despite ECMO administration, and expired in the ICU with comfort measures only a few weeks after the event.

**Figure 2:**
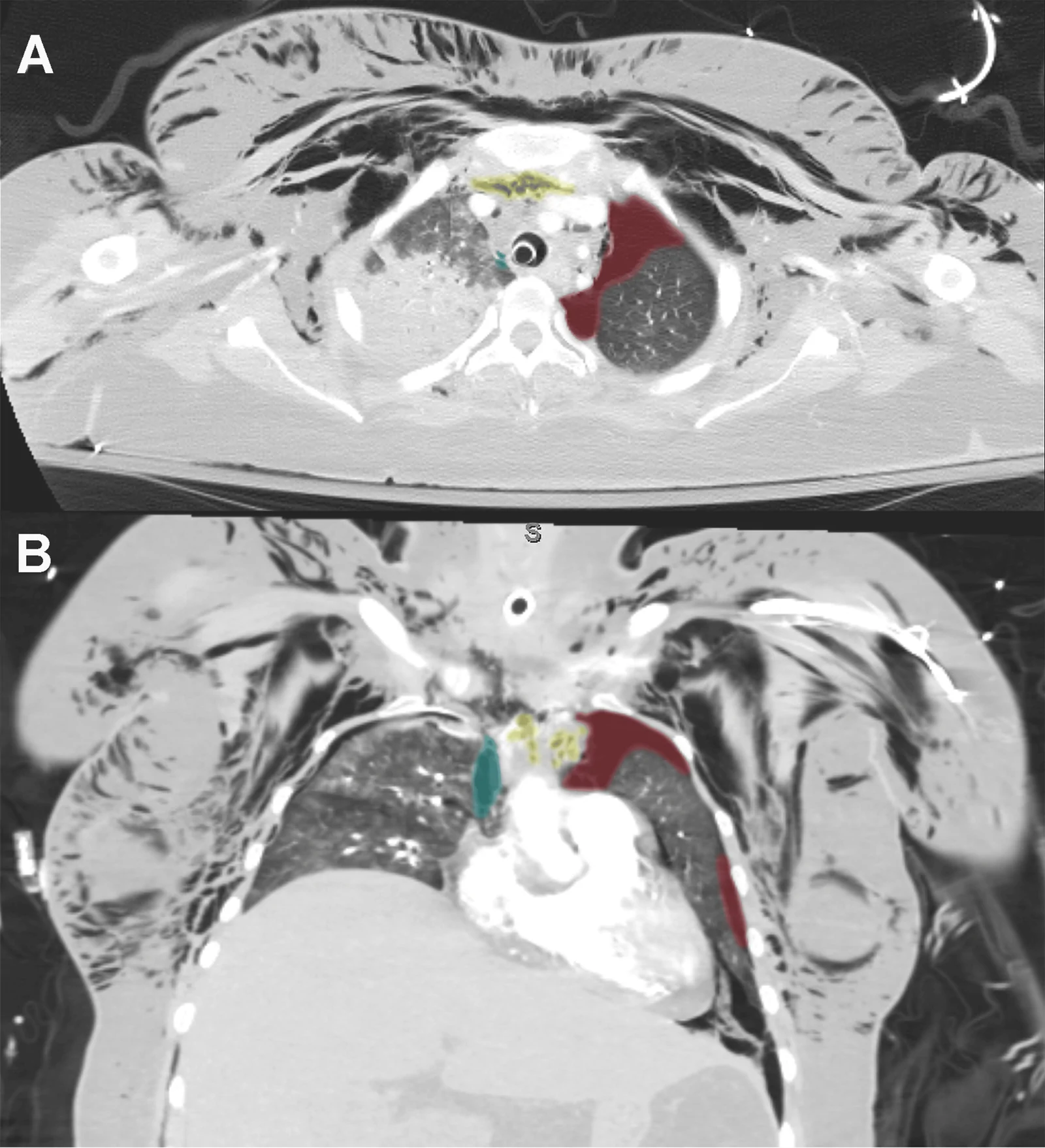
CT of the chest with angiography showing soft-tissue emphysema, pneumomediastinum, and pneumothorax. (A) Axial CT of the chest with angiography showing right lobar aspiration pneumonia, soft-tissue emphysema (yellow), left pneumothorax (red), and pneumomediastinum (blue) from suspected false passage. (B) Coronal CT of the chest with angiography soft-tissue emphysema (yellow), left pneumothorax (red), and pneumomediastinum (blue) from suspected false passage.

## Discussion

This case underscores the risk of false passage, leading to difficult airway management in a patient with a history of tracheostomy, particularly when the tracheostomy tube had been recently removed. Despite the use of ECMO in this case, the lack of a readily available FOB at our facility delayed management of a difficult airway, while anchoring bias toward presumed bronchospasm further prolonged the appropriate diagnosis.

Fiberoptic bronchoscopy: a critical solution

While the literature on intubation post-tracheostomy is limited, cases involving airway management after blunt trauma suggest improved outcomes with the use of FOB for airway visualization [11-14]. FOB’s ability to confirm correct ETT positioning and guide necessary adjustments makes it an essential tool in difficult airway management, particularly in patients with a history of tracheostomy [15]. A recent randomized controlled trial showed significant improvements in first-pass intubation, intubation time, and positioning time when visual laryngoscopy was complemented with the use of FOB [3]. In our facility, FOB access is limited, even in operating rooms, and its immediate access could have facilitated earlier recognition of the false passage airway through direct visualization of the tracheal wall during intubation, minimized delays in ventilation, and reduced the risk of hypoxic injury [9].

A literature search of PubMed and Web of Science conducted on March 30, 2024, using the key terms “tracheostomy” AND (“false passage” OR “false airway”), did not identify any other reported cases of a false-passage airway occurring after visual evidence of cutaneous healing of a tracheostomy stoma and passage of an ETT through the vocal cords and larynx. While the most common airway concern post-tracheostomy is tracheal stenosis, this case demonstrates that less common complications, such as false-passage airways, can occur and may necessitate rapid intervention with advanced tools like FOB [9]. Upon careful review of this case, easy access to FOB may have facilitated faster recognition of the unexpected non-healed tracheostomy, reducing time to effective ventilation and potentially preventing anoxic brain injury.

Addressing cognitive bias

In addition to the absence of FOB, cognitive biases, particularly anchoring bias, may have contributed to delays in recognizing the true cause of the ventilation failure. Anchoring bias, fixating on bronchospasm as the diagnosis, may lead clinicians to overlook alternative explanations, especially in time-sensitive situations [6]. Studies have demonstrated that cognitive biases can affect decision-making in over 50% of observed scenarios, underscoring their impact on clinical outcomes [5].

Cognitive aids, such as checklists, and fostering team-based decision-making can help mitigate the effects of biases, enabling clinicians to respond more effectively to unexpected complications [5,6]. This case highlights the importance of maintaining a broad differential to avoid anchoring bias.

A unified approach

The interplay between advanced tools such as FOB, ECMO, and strategies to address cognitive biases is key to managing complex airway scenarios. Established algorithms remain instrumental in reducing adverse outcomes but are not infallible [4]. Clinicians must adopt a dual approach: ensuring critical equipment is available and fostering a culture of vigilance and adaptability in clinical decision-making.

Proposed solutions to improve patient outcomes include increasing the availability of FOB and ECMO in clinical settings, particularly for managing unanticipated difficult airways, and enhancing provider education on recognizing and mitigating cognitive biases. Incorporating FOB into early difficult airway management protocols enhances visualization, supports precise tube placement, and facilitates timely intervention, especially when ECMO is not readily available, optimizing patient care and reducing the incidence of adverse outcomes [3].

At present, FOB availability has improved at our facility, particularly in specialized areas such as operating rooms and ICUs. Potential future efforts may include targeted workshops, simulation-based training for difficult airway management, and advocacy for procuring additional FOB units in high-risk areas. These steps align with broader institutional goals of improving patient safety and airway management outcomes.

This case underscores the critical importance of FOB in managing unexpected airway complications, particularly in patients with prior tracheostomies. The availability of FOB enables precise airway assessment and timely intervention, reducing the risk of adverse outcomes. Additionally, recognizing and mitigating cognitive biases, such as anchoring bias, can enhance clinical decision-making and minimize delays in appropriate action. A dual focus on equipping teams with advanced tools and fostering cognitive awareness is essential for improving patient outcomes in challenging airway management scenarios.

## Conclusions

This case underscores the critical importance of FOB in managing unexpected airway complications, particularly in patients with prior tracheostomies. The availability of FOB enables precise airway assessment and timely intervention, reducing the risk of adverse outcomes. Additionally, recognizing and mitigating cognitive biases, such as anchoring bias, can enhance clinical decision-making and minimize delays in appropriate action. A dual focus on equipping teams with advanced tools and fostering cognitive awareness is essential for improving patient outcomes in challenging airway management scenarios.
